# Sequestration of steroidal estrogen in aqueous samples using an adsorption mechanism: a systemic scientometric review†

**DOI:** 10.1039/d3ra02296j

**Published:** 2023-07-26

**Authors:** Ajibola A. Bayode, Chijioke Olisah, Stephen Sunday Emmanuel, Morenike Oluwabunmi Adesina, Daniel Terlanga Koko

**Affiliations:** a Department of Chemical Sciences, Faculty of Natural Sciences, Redeemer's University P.M.B. 230 232101 Ede Nigeria bayodea@run.edu.ng; b Institute for Coastal and Marine Research, Nelson Mandela University P. O Box 77000 Gqeberha 6031 South Africa; c Department of Industrial Chemistry, Faculty of Physical Sciences, University of Ilorin P.M.B. 1515 Ilorin Nigeria; d Department of Chemistry, Faculty of Natural and Applied Sciences, Lead City University Nigeria

## Abstract

Steroidal estrogens (SEs) remain one of the notable endocrine disrupting chemicals (EDCs) that pose a significant threat to the aquatic environment in this era owing to their interference with the normal metabolic functions of the human body systems. They are currently identified as emerging contaminants of water sources. The sources of SEs are either natural or synthetic active ingredients in oral contraceptive and hormonal replacement therapy drugs and enter the environment primarily from excretes in the form of active free conjugate radicals, resulting in numerous effects on organisms in aquatic habitats and humans. The removal of SEs from water sources is of great importance because of their potential adverse effects on aquatic ecosystems and human health. Adsorption techniques have gained considerable attention as effective methods for the removal of these contaminants. A systemic review and bibliometric analysis of the application of adsorption for sequestration were carried out. Metadata for publications on SE removal utilizing adsorbents were obtained from the Web of Science (WoS) from January 1, 1990, to November 5, 2022 (107 documents) and Scopus databases from January 1, 1949, to November 5, 2022 (77 documents). In total, 137 documents (134 research and 4 review articles) were used to systematically map bibliometric indicators, such as the number of articles, most prolific countries, most productive scholars, and most cited articles, confirming this to be a growing research area. The use of different adsorbents, include activated carbon graphene-based materials, single and multi-walled carbon nanotubes, biochar, zeolite, and nanocomposites. The adsorption mechanism and factors affecting the removal efficiency, such as pH, temperature, initial concentration, contact time and adsorbent properties, were investigated in this review. This review discusses the advantages and limitations of different adsorbents, including their adsorption capacities, regenerative potential, and cost-effectiveness. Recent advances and innovations in adsorption technology, such as functionalized materials and hybrid systems, have also been highlighted. Overall, the bibliographic analysis provides a comprehensive overview of the adsorption technique for the removal of SEs from other sources, serving as a valuable resource for researchers and policymakers involved in the development of efficient and sustainable strategies to mitigate the effects of these emerging contaminants.

## Abstract 2

Steroidal estrogens (SEs) pose a significant threat to the aquatic environment in this era because they interfere with the human body's normal metabolic functions, this calls for their sequestration.

## Graphical abstract

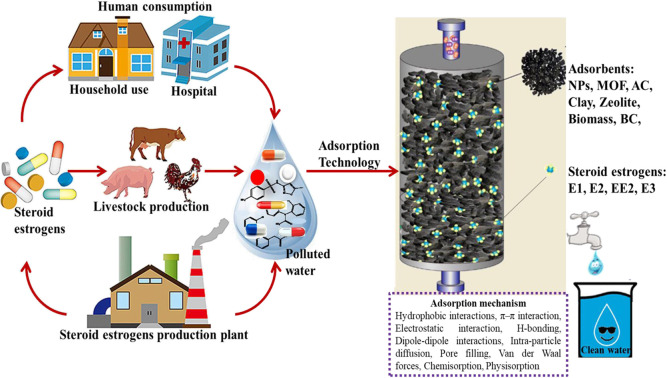

## 1. Introduction

The persistent increase in anthropogenic activities has led to the indiscriminate release of chemicals that contaminate waterbodies and negatively affect the aquatic ecosystem owing to high toxicity and persistence in the environment.^1,2^ These chemicals are of natural and synthetic origin and include endocrine disrupting chemicals with concentrations ranging from ng L^−1^ to g L^−1^ in aquatic bodies.^3,4^

“Endocrine disrupting chemicals according to the definition by the World Health Organization (WHO) are chemicals that interfere with the normal function of humans and wildlife endocrine system by blocking or mimicking the way hormones control metabolism, growth, and body function”. EDCs include polyfluorinated alkyl,^5,6^ pharmaceuticals,^7–10^ steroid estrogens,^2,11^ pesticides,^12^ and personal care products.^13,14^

Over the past few decades, the occurrence of steroid estrogens (SEs), a class of emerging contaminants in the environment and aquatic environment, has been of serious concern because of rapid urbanization and industrialization,^2,15^ posing deleterious health challenges and environmental concerns, endangering all forms of life due to the persistent, toxic and estrogenic nature of EDCs.^16–19^

Steroid estrogens are a group of hormones classified as naturally occurring and synthetically developed. The naturally occurring SEs are estrone (E1), 17-β-estradiol (E2), and estriol (E3), which play a vital role in acting as the female sex hormone responsible for the development and regulation of the female reproductive system and the secondary sex character,^4,20^ while the synthetically developed estrogen 17-α ethinylestradiol (EE2) is the active ingredients in oral contraceptive and hormone replacement therapy drugs, which is approved for use in the treatment of menopause symptoms, hypoestrogenism and prevention of osteoporosis by the food and drug administration (FDA).^4,21^

Steroid estrogens enter the environment *via* different pathways. Humans and animals largely excrete these estrogens *via* urine and faeces as active free forms of glucuronide conjugate and sulfate conjugates, which are deconjugated back in the environment or in their original form as the body metabolizes only 24–48% of the oral contraceptive dose in the body. Animals excrete more steroid estrogen than humans.^4,20^ Other sources of steroid estrogens in the environment include animal waste, run offs from farms and effluents from wastewater treatment plants^3,4,20,22^

**Fig. 1:**
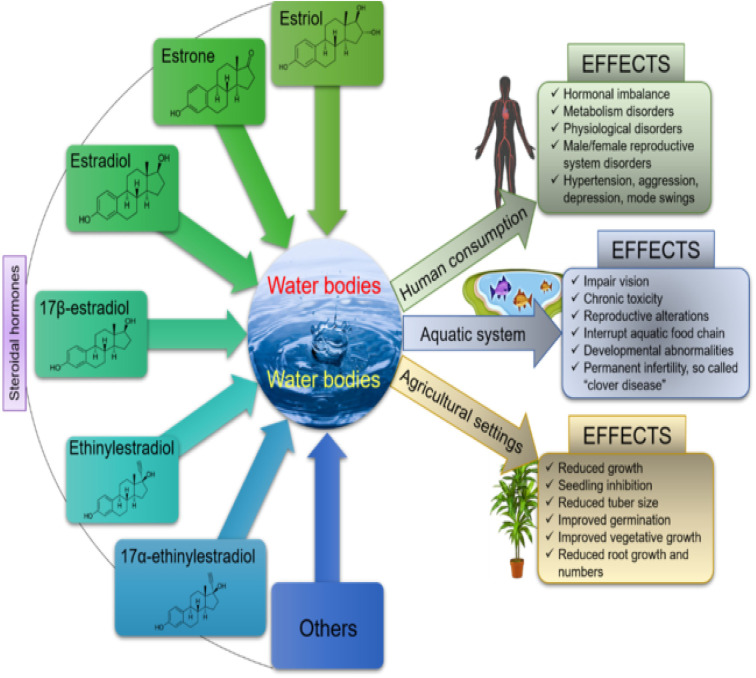
Schematic representation showing the effect of SEs on humans, plants and aquatic life^22^ (open access).

Steroid estrogens are known to have a negative effect on human and aquatic life forms when present even at very low concentrations.^2,18,20^ They have been linked to an increase in testicular, breast, and ovarian cancer, obesity, infertility, hormonal imbalance, low sperm count in adult males, fibroid, and endometriosis in adult females.^3,23,24^ In aquatic animals, it causes feminization of the fishes, impaired vision, and clover disease, while in plants, it causes reduced growth and seeding inhibition,^25,26^ as shown in Fig. 1. Therefore, water treatment has become a critical issue globally, which is essential for improving the stability of ecology and human health.

To sequestrate these SEs in water, the conventional water treatment system cannot cater for their removal^2,20^ because many of the chemicals have not been included in the wastewater treatment legislation (Directive 2000/60/EC, Directive 2008/56/EC, and Directive 2013/39/EU),^27,28^ making researchers explore other types of treatment technologies that have been developed. Different types of treatment technologies have been developed, such as advanced oxidation processes,^15^ chlorination,^29^ coagulation–flocculation,^30^ electrocatalysis,^31^ photocatalysis,^2,20^ adsorption,^11,18,32^ membrane technology,^33,34^ and reverse osmosis.^35,36^ The major shortcomings of these technologies are the high cost of operation and the production of secondary by-products, which may be more toxic and complex.^37,38^

According to research reported by various scientists over the years, adsorption techniques have proven to be one of the most promising approaches for removing SEs from water because they are cheap, easy, environmentally benign and do not lead to the production of secondary by-products.^27,38^

This paper presents a review of the use of adsorption technology for the removal of SEs from water; it comprehensively examines the old status, current status and the future of this research topic through a detailed systemic review and large-scale bibliometric analysis performed based on information search on the ISI web of science search engine using the keywords “steroid estrogen (s)”, “removal”, “adsorbents”, article citation, author's collaboration, country, annual outputs, *etc.*, with the majority of published research works spanning the last 50 years (1973–2022).

## 2. Chemistry of estrogen and reactions in water

**Fig. 2:**
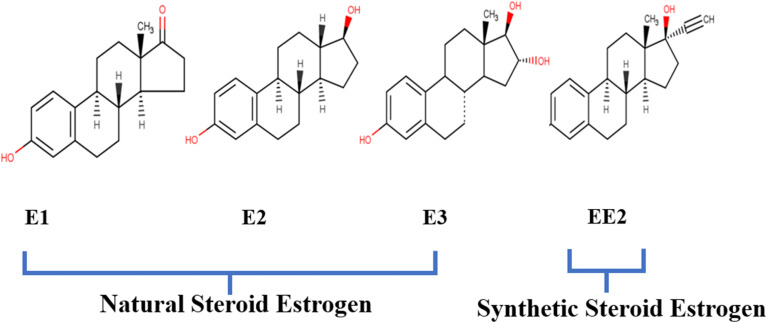
Structure of SEs^20^ (open access).

The term “estrogens” refers to a group of biologically active hormones formed in humans and animals by the testes, adrenal cortex, placenta, and ovary.^39^ They belong to a class of steroid chemicals known as major female sex hormones because of their significance in the estrus cycle. Steroid estrogens can be classified as either natural or synthetic hormones, and when present in excess in the living organism, they can serve as endocrine disruptors (EDCs).^40^ The cyclopentane phenanthrene ring serves as the building block for the chemical structure of estrogens, as shown in Fig. 2. The additional carbon contributes to the formation of the estran (C18) structure.^41^

Natural estrogens, commonly known as the C18 steroidal group, have four rings: one cyclopentane, two cyclohexanes and one phenolic ring.^42^ They regulate the development of secondary female sex traits and, in conjunction with the gestagens, regulate all reproductive processes in women. Natural estrogens have different amounts and configurations of hydroxyl groups, such as estrone (E1), 17β estradiol (E2), estriol (E3), and estetrol (E4).^43^ Synthetic estrogens, however, are a pharmaceutical substance used as an oral contraceptive and hormonal replacement therapy. They are also utilized to stimulate animal growth. 17-ethinyloestradiol is the most commonly known synthetic estrogen.^44^

To determine the fate of steroidal estrogen chemicals in soil and water systems, it is essential to understand their physiochemical characteristics. The dispersion of organic contaminants in the aqueous phase and other solids is frequently regarded as a function of the partitioning between the organic and aqueous phases.^45^ The proportion of a compound's concentration under equilibrium conditions in *n*-octanol and water at a specific temperature is known as water partition coefficient (*K*_ow_).^45^ Large molecules and compounds with a high log *K*_ow_ > 5 are easily adsorbed to sediments and are mostly removed by coagulation. Because estrogens have a substantial log *K*_ow_, it is expected that they are absorbed onto the solid phases.^45^

In general, free or unconjugated estrogens are not highly water soluble. Most SEs are usually moderately hydrophobic (log *K*_ow_ = 2.4–4.0); E2 is the most hydrophobic of the estrogens, whereas EE2 is the least soluble. At neutral pH, aqueous solubility progresses from E1 (one OH group) to E2 (two OH groups), and then EE2 with the addition of ethinyl groups at the 17-position on the D ring; solubility appears to be the same at pH 4.^45,46^ However, solubility can be affected by pH because, for instance, estrogen's relative solubility is higher at pH 10.^47^

Estrogens are hydroxylated in the liver. The conversion of the hydroxyl (OH) groups into =O results in the transformation of E2 to E1, which is further converted to E3 because of subsequent changes.^48^ Subsequently, sulphate and glucuronic derivatives are produced after esterification, which is later excreted with urine or bile. Most often, estrogens eliminated with bile are partly reabsorbed in the colon, while the remaining substances are expelled from the body system with faeces;^45^ they enter the natural environment alongside excrement.^49^

SEs are not volatile and are therefore relatively short-lived in the environment. Consequently, they are unlikely to penetrate the atmosphere in considerable quantities and to be transported a great distance from the place of emission.^50^

## 3. Methodology of data retrieval and processing

We retrieved metadata on SE removal using adsorbents from the Web of Science (WoS) and Scopus databases. The former is hosted on the Clarivate Analytics platform, a leading citation database. The WoS contains a diverse range of multidisciplinary literature, particularly those related to biological, physical, and life sciences, where the theme of the current study lies.^51^ Inaugurated in 2004 by Elsevier, the Scopus databases represent a comprehensive information system with exhaustive citation metrics and abstracts of a range of disciplines.^52^ Adopting the guideline stipulated in Olisah *et al.* (2022), we mapped out research trends from retrospective encoded data on SE removal using adsorbents from the WoS and Scopus databases. Only studies related to the research areas were retrieved. To identify studies conducted on SE removal using adsorbents, a search string TI = (“estrogen”) AND TI = (removal) AND TI = (“adsorbent*) was used to retrieve articles indexed in the WoS database from January 1, 1990, to November 5, 2022. Only “Article” (*n* = 103), “Review Article” (*n* = 3) and “Book Chapters” (*n* = 1) were targeted. Document types, such as “Proceeding Papers” (*n* = 3) and “Early Access” (*n* = 2), were excluded. After exclusion, a total of 107 documents were identified. Similar search terms were inserted into the TITLE-ABS-KEY Scopus database module focusing on “Article” (*n* = 74), “Review” (*n* = 2), and “Book Chapter” (*n* = 1) published from January 1, 1949, to November 5, 2022, while “Conference Paper” (*n* = 3) was excluded. This yielded a total of 77 articles.

A total of 107 WoS and 77 indexed Scopus documents were downloaded in Bibtex format and uploaded into the RStudio application (Version 1.4.1106; 2009–2021) for bibliometric processing. Duplicate documents from both databases were combined as one with the R code “h < -duplicated Matching (M, Field = “TI”, tol = 0.95”), giving a total of 137 documents that were used to systematically map bibliometric indicators, such as the number of articles, most prolific countries, most productive scholar, and most cited articles. All codes for statistical bibliometric and statistical analysis (Kolmogorov–Smirnoff (K–S) *p*-value, goodness of fit, and β-coefficient) were adopted from Aria and Cuccurullo (2017). Bibliometric coupling was done using the equation *D* = *C* × *CT*, where C represents the bipartite network and *D* represents the symmetrical matrix. Two articles are considered bibliometrically coupled if a cited reference is shown in both articles.^53^ Country collaborations were mapped using the following set of parameters: *n* = 2 and label size = 14. Combined metadata from the WoS and Scopus databases were downloaded in CSV format and uploaded on the VoS Viewer App (version 1.6.15 © 2009–2020) for thematic classification using the keyword plus and author's keywords. We used linear and polynomial models to analyse research trends; however, we chose the latter as a predictive growth model owing to its reasonable *R*^2^ value.^54^

### 3.1 Publication trends

**Fig. 3:**
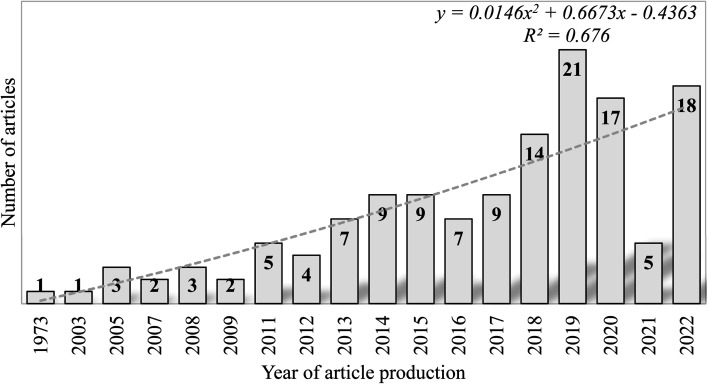
Annual publication frequencies of research involving the removal of SEs from the environment published from 1949 to 2020.

A total of 137 articles were retrieved from the merged databases from 1949 to 2022 (Table S1†), of which 133 were research articles and four were review articles. The studies were conducted by 476 authors with document/author, author/document, and co-author/document ratios of 0.29, 3.47, and 4.91, respectively. All publications were multiple authored and retrieved from 75 journal sources. The retrieved documents in the research focus area accumulated an average citation/document and average citation per year of 5.51 and 3.69, respectively, with a collaboration index of 3.47. The first publication on SE removal was published in 1972, and document production from then until 2009 was relatively steady, with only six years recording at least one publication (1973, 2003, 2005, 2007, 2008, and 2009), as shown in Fig. 3. Peak numbers were recorded in 2019 with 21 articles, followed by 2020 and 2022 with 17 and 18 articles, respectively.

The presence of endocrine-disrupting chemicals in our environment has become a global menace because of their potential to affect terrestrial and aquatic lifeforms adversely. Many of these EDCs are plasticizers, polyestrogens, pesticides, and pharmaceuticals. Emerging contaminants, such as synthetic SEs (17-α ethinyl estradiol – analogue of natural estrogen – 17β-estradiol), have received significant interest from scholars owing to their persistent nature in the environment. This compound, together with estrone and estriol, has been confirmed to be the most potent and ubiquitous environmental estrogen present in various female contraceptives and hormone replacement therapy.^55^ Our studies revealed that about 88% of most articles on SE removal have been published in the last decade. In this era, researchers were beginning to understand only the adverse effects of these compounds and to explore scientific methods for their removal. Many international initiatives and funding schemes inaugurated to tackle water pollution may be responsible for the high research output in the past few decades. These include the National Institute of Food and Agriculture National Integration Water Quality Program, which was established in 2015 to improve water quality across the United States (https://www.nifa.usda.gov/), and the approval of USD 114 million by OPEC in 2020 for the water and sanitation sector in Africa, Europe, Asia, Latin America and the Caribbean (https://opecfund.org/focus-areas/water-sanitation). The adoption of the Sustainable Development Goals (SDG) by the world's leaders, particularly those linked to water pollution (SDG 16 and SDG 14), may also have spurred research in this area in the last decade. The association between the number of articles and the year of production fitted into a polynomial model, which generated an *R*^2^ of 0.7, thus indicating a strong relationship between both indices. We employed Lotka's inverse square law of author predictive to assess authorship distribution dynamics.^56^ Coupled with other statistical tools (Kolmogorov-Smirnoff goodness of fit = 0.93, *p* > 0.01 and β-coefficient = 2.35), Lotka's distribution revealed that Lotka's law does not fit the literature of SE removal. An annual growth rate of 6.07% further suggests that a slow output of literature in this research area is likely in subsequent years.

### 3.2 Most productive countries and citation analysis

**Fig. 4:**
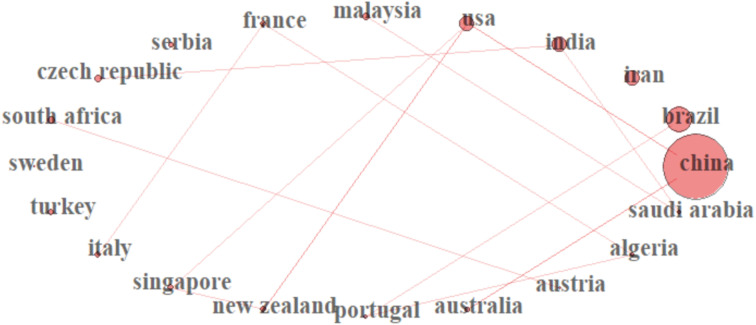
Corporation network of 20 of the most productive countries on research on SE removal.

This study prioritizes this bibliometric indicator to identify the most productive countries in the considered subject area. As shown in Table S2,† countries were ranked based on the number of articles and the citation metrics produced by authors affiliated with the institutions of these countries. China had the highest publications (*n* = 47), accounting for 34.3% of the total articles, followed by Brazil (*n* = 17; 12.4%), Iran (*n* = 11; 8.03%), India (*n* = 8; 5.84%) and the USA (*n* = 6; 4.38%). China also topped the chart on citation metrics with 1172 citations, followed by the USA (*n* = 442), Spain (*n* = 357), India (*n* = 342), and the United Kingdom (*n* = 325). It is important to note that 50% of the top countries in the research on SE removal are developed countries. The presence of cutting-edge facilities, scientific advancement, government involvement in scientific research, and adequate funding may be responsible for their presence. With population growth and economic progress, China has gradually become one of the largest users of pharmaceuticals.^57^ Various SE compounds have been widely detected in their environments, and their removal has been deeply concerned by researchers.^58,59^ In China, over 50 000 of 36 different pollutants contaminated the environment in 2013, and in 2015, the State Council of China established the “Water Pollution Prevention and Control Action Plan” to tackle the water pollution crisis in the country.^60,61^ The position of China in the number one spot may also be attributed to the efforts made by the Chinese government to remove pharmaceuticals.^62^ The findings of this study align with a similar study that also ranked China first when it comes to eliminating pharmaceutical and personal care products (PPCPs). The importance of collaboration in scientific research cannot be over-emphasized as it fosters research progression and makes actualizing a set of research objectives easier.^63^Fig. 4 depicts the corporate network of 20 of the most productive countries in the subject area. The line thickness between countries reflects the collaboration frequencies, while the red circle nodes show the number of countries. China had the highest collaboration strength, mainly with the USA and Australia, followed by Brazil, the USA, and India.

Citation analysis was prioritised in this study to identify the most influential articles among all documents indexed in the WoS and Scopus databases. The top 10 most cited articles are listed in Table S3.† Pan *et al.* (2018) topped the retrieved records with “Adsorption and hysteresis of bisphenol A and 17α-ethinyl estradiol on carbon nanomaterials”, accumulating a total citation of 366 with an average citation rate of 24.4% per year.^64^ This was followed by “Removal of estrone and 17β-estradiol from water by adsorption” and “Determining estrogenic steroids in Taipei waters and removal in drinking water treatment using high-flow solid-phase extraction and liquid chromatography/tandem mass spectrometry” in the second (*n* = 172; 9.56%) and third (*n* = 151; 9.44%) spots, respectively.

### 3.3 Classification of thematic areas based on keyword occurrences

**Fig. 5:**
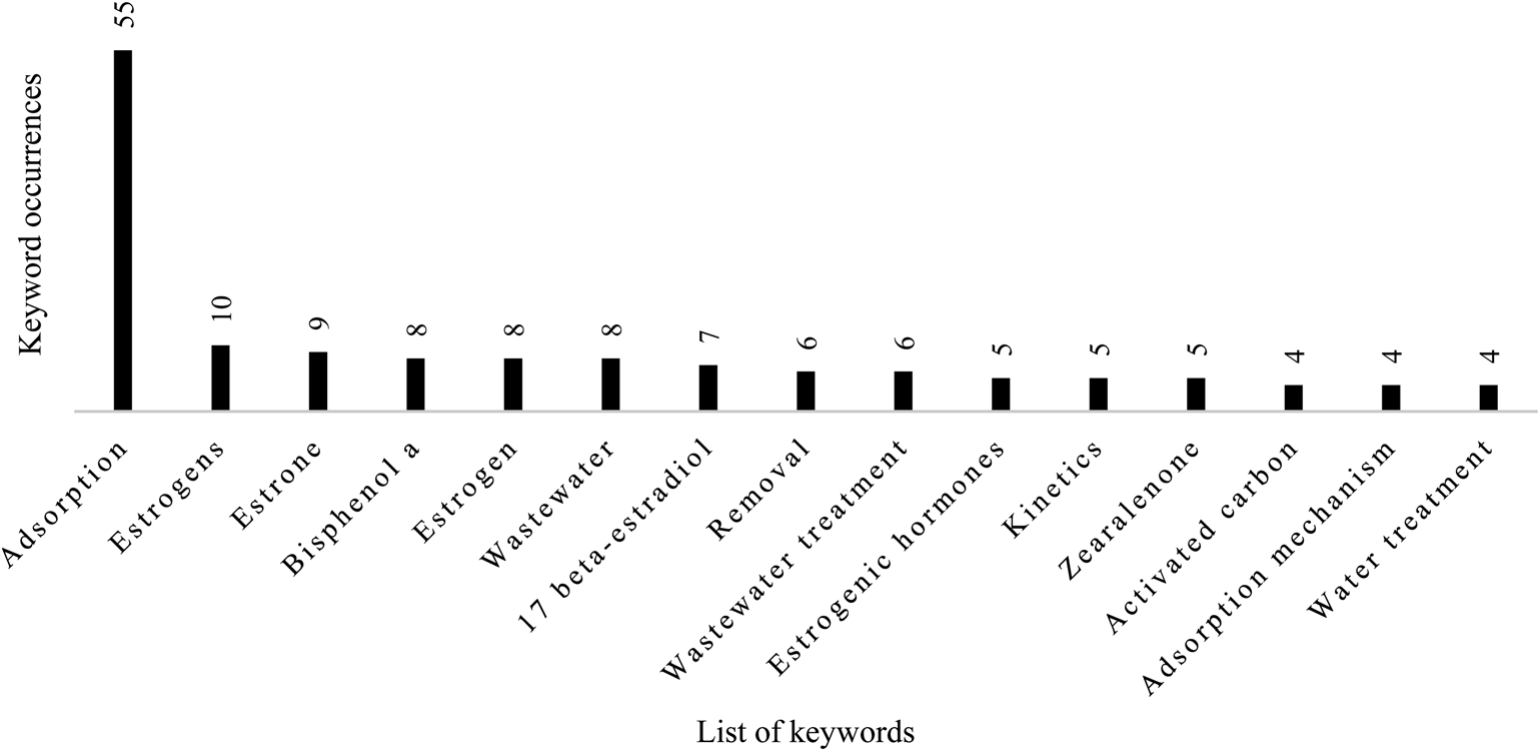
Frequency of the 15 most used keywords in articles associated with SE removal.

Keywords represent one of the most essential contents during the submission of a manuscript for publication. Categorizing and analysing keywords can effectively capture the research area most important to researchers. The analysis of the occurrence of keywords has become a common practice when performing bibliometric studies. A total of 417 authors' keywords were found in the 137 articles retrieved for this study, thus reflecting a diversified author preference. Fig. 5 displays the top 15 keywords used in articles published from 1949 to November 2022. Adsorption, removal, and estrogens are the words used to retrieve articles in the hybrid database, so they can be ignored. As shown in Fig. 4, keywords in the top 15 are “estrone” (*n* = 9, 2.16), “Bisphenol A”, “wastewater” (*n* = 8, 1.92%), “17 beta-estradiol” (*n* = 7, 1.68), “wastewater treatment” (*n* = 6, 1.44%), and “estrogen hormones” “kinetics”, and “zearalenone” (*n* = 5, 1.20%). Others include “activated carbon”, “adsorption mechanism” and “water treatment” (*n* = 4, 0.96%).

**Fig. 6:**
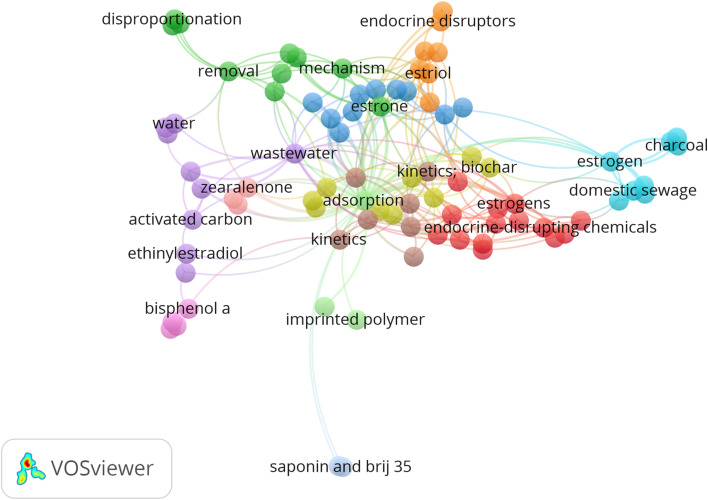
Co-occurrence network of the most frequently used keywords in articles associated with SE removal.

As shown in Fig. 6, imprinted polymer, biochar, and activated carbon/charcoal are the commonly used adsorbents for removing SEs from contaminated water. In general, activated carbon/charcoal has become the most widely used adsorption material for removing pharmaceuticals owing to its large surface area, chemical stability, and developed physical structure.^65^ This material's low cost and high removal efficiency properties may be responsible for its frequent usage in the treatment of water contaminated with SEs.

## 4. Adsorption technique for the removal of SEs in water

**Fig. 7:**
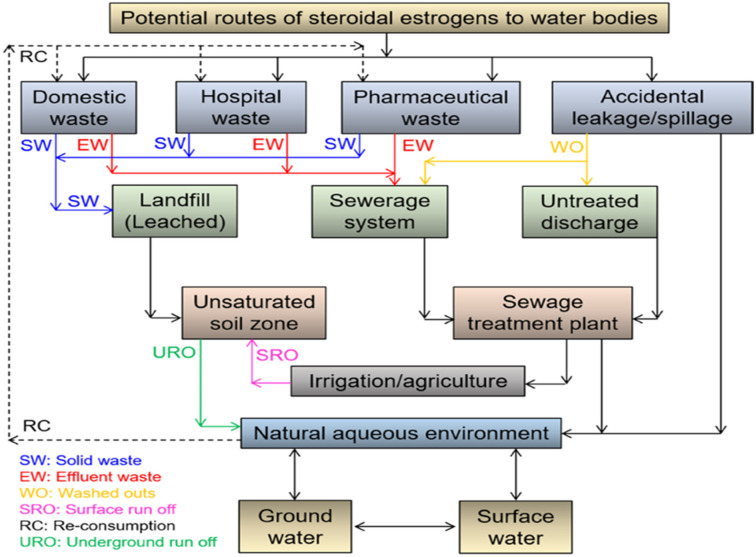
Sources and routes of SE contamination in the aquatic system.^70^ Reused with permission from Elsevier (order no: 5577471429890).

Over the years, steroidal estrogens (SEs) have been considered widespread water contaminants, as shown in Fig. 7, and the most lethal kind of EDC owing to their strong affinity for nuclear receptors;^18,66,67^ thus, their remediation from the aquatic ecosystem is of primary and growing concern owing to their deadly effect on creatures and eco-networking.^18,68^ Notably, SEs have been labelled as contaminants of emerging concern because they are popularly recognized to engender deleterious changes in marine habitats when present even at relatively close-to-zero concentrations.^18,66,68–72^

Interestingly, in recent times, different functional materials, such as MOF, nano adsorbents, unmodified biomass, activated carbon (AC), clays, and biochar (BC), have been used as adsorbents for the adsorptive removal of various types of SE in water. The pragmatic efficiency of these materials in recently conducted SE's adsorption studies is holistically discussed in this section based on their percentage efficiency and adsorption capacity, which were approximated to two decimal points, as depicted in Table 1.

**Table 1:** Adsorption capacity of various functional adsorbents for the removal of different SEs^a^

Adsorbent	ASE	HAC (mg g^−1^)	HAE (%)	OP	OT (°C)	AT (min.)	Adsorption mechanism	Best fit isotherm	Best fit kinetics	References
AC-alginate biopolymer	EE2	0.53	84.00	3	—	30	—	FI	—	86
Fe_3_O_4_@SiO_2_@MPS	E2	27.35	92.10	6	30	120	H-Bonding	FI	PFO	74
Rice husk silica	E1	0.56 × 10^−4^	93.10	4	—	60	—	LI	PFO	82
	E2	0.62 × 10^−4^	95.50	4	—	60	—	LI	PFO	
GO-magnetic rice straw BC	E2	46.22	—	7	25	1200	H-Bonding, electrostatic and π–π interactions, chemisorption	FI	PSO	94
Mg_3_Al-layered double hydroxide intercalated with Na-dodecyl sulfate	E2	—	94.00	7	25	45	Hydrophobic interactions, and H-bonding	FI	PSO	83
Rice husk	E1	2.70	>98	7	25	60	H-Bonding	LI	PFO	85
	E2	1.65	>98	7	25	120	H-Bonding	LI	PSO	
									PFO	
	E3	1.00	>77	7	25	120	H-Bonding	LI	PFO	
Rice husk	E1*	2.64	—	7	25	60	H-Bonding	LI	PFO	85
	E2*	1.59	—	7	25	120	H-Bonding	LI	PFO	
	E3*	0.89	—	7	25	120	H-Bonding	LI	PFO	
*N*-Propyl-mesoporous silica	E1	88.38	>70	7	25	60	Hydrophobic, hydrogen-bonding, and dipole–dipole interactions	LI	PSO	96
	E2	86.91	70	7	25	120		LI	PSO	
	EE2	119.87	>80	7	25	120		LI	PSO	
β-Cyclodextrin polymers	E2	126.16	>99	7	25	—	Hydrophobic, and hydrogen-bonding interactions	LI	PSO	97
	EE2	145.81	>99	7	25	—	Hydrophobic, and hydrogen-bonding interactions	LI	PSO	
γ-Cyclodextrin polymers	E2	198.46	>99	7	25	—	Hydrophobic, and hydrogen-bonding interactions	LI	PSO	97
	EE2	216.87	>99	7	25	—	Hydrophobic, and hydrogen-bonding interactions	LI	PSO	
Poly(HEMA-MAPA) microparticles	E2	98.4	>90	—	25	150	Hydrophobic interactions and physisorption	LI	—	98
BC pellet	E1	6.39		55	25	15	Hydrophobic interactions and chemisorption	FI	PSO	99
	E2	10.43		5	25	15	Hydrophobic interactions and chemisorption	FI	PSO	
	E3	4.80		5	25	15	Hydrophobic interactions and chemisorption	FI	PSO	
Soybean hulls	E1	2.59	100	7	25	60	H-bonding	LI	PFO	100
							Physisorption			
							Hydrophobic interactions			
	E2	2.24	100	7	25	120	H-bonding	LI	PSO	
							Physisorption			
							Hydrophobic interactions			
	E3	0.86	100	7	25	120	H-Bonding	LI	PFO	
							Hydrophobic interactions		PSO	
							Physisorption			
Soybean hulls	E1*	2.56	100	7	25	60	H-Bonding	LI	PFO	100
							Hydrophobic interactions			
							Physisorption			
	E2*	1.98	100	7	25	120	H-Bonding	LI	PFO	
							Hydrophobic interactions			
							Physisorption			
	E3*	0.84	100	7	25	120	H-Bonding	LI	PFO	
							Hydrophobic interactions			
							Physisorption			
*Macadamia* nutshell AC	E1	22.00	85	7	25	120	H-Bonding	LI	PSO	102
							Physisorption			
							Hydrophobic interactions			
							Chemisorption			
							Electrostatic interactions			
	E2	22.00	82	7	25	120	H-bonding	LI	PSO	
							Electrostatic interactions			
							Physisorption			
							Hydrophobic interactions			
							Chemisorption			
Mesoporous imprinted polymer	E1	371.20	100	9	—	20	Intra-particle diffusion	LI	PSO	91
							Chemisorption			
	E3	323.10	100	9	—	20	Intra-particle diffusion	LI	PSO	
							Chemisorption			
Mesoporous non-imprinted polymer	E1	285.30	100	9	—	20	Intra-particle diffusion	LI	PSO	91
							Chemisorption			
	E3	237.40	100	9	—	20	Intra-particle diffusion	LI	PSO	
							Chemisorption			
Boron nitride nanosheets	E1	249.15	96.34	—	25	—	Hydrophobic, and π–π interactions	LI	PSO	103
							van der Waal's			
Yeast biomass	E1	0.93	>90	10	—	20	Electrostatic interactions	FI	PSO	106
	EE2	21.41	>90	10	—	20	Electrostatic interactions	FI	PSO	
Modified zeolites	E1	41.67	—	5	25		Distribution effects and surface adsorption	LI	PSO	107
	E2	23.87	—	5	25	—	Distribution effects and surface adsorption	LI	PSO	
Thermally activated sludge	E2	8.75 × 10^−3^	—	5.5	—	360	Chemisorption	FI	Elovich	114
	EE2	14.56 × 10^−3^	—	5.5	—	240	Chemisorption	LI/FI	PFO	
KOH activated sludge	E2	17.90 × 10^−3^	—	5.5	—	240	Chemisorption	LI/FI	PFO	114
	EE2	0.44 × 10^−3^	—	5.5	—	360	Chemisorption	LI	Elovich	
H_2_SO_4_ activated sludge	E2	16.42 × 10^−3^	—	5.5	—	360	Chemisorption	LI/FI	PFO	114
	EE2	4.23 × 10^−3^	—	5.5	—	420	Chemisorption	LI	Elovich	
Fe mining tailing CNT	EE2	21.60	—	6	25	120	π–π interactions	FI	PSO	118
Corn straw BC	EE2	1.70	—	7	25	480	Chemisorption	FI	PSO	117
							H-Bonding			
Mesoporous carbons	EE2	157.00	99	7	—	45	Physisorption	FI	PSO	109
Plastic-char	EE2	82.30	—	7	25	600	Covalent and π–π interaction	FI	PSO	115
							Chemisorption			
Attapulgite	E2	28.12	—	3.5	25	—	H-Bonding	LI	PSO	116
							Chemisorption			
							Electrostatic and π–π interaction			
Sulfur-doped g-C_3_N_4_	E2	33.38	100	7	25	45	—	—	PSO	130
Walnut shell BC	E1	21.36	>45	4	25	120	—	FI	PSO	111
	E2	17.74	50	4	25	120		FI	PSO	
	E3	25.07	40	4	25	120		FI	PSO	
Uncarbonized Fe/Ni NPs (using *eucalyptus* leaf extract)	E2	35.00	77.60	6	40	30	H-Bonding	FI	PSO	127
							π–π interaction			
							Intraparticle diffusion			
Fe_3_^+^-saturated montmorillonite	E2	—	93.00	6	40	30	—	—	—	119
FeNPs/rGO (using green tea extract)	EE2	11.34	56.10	6.5	40	—	π–π interaction	LI	PSO	131
									PFO	
rGO@Fe NPs (using green tea extract)	EE2	—	99.90	6	40	—	—	—	—	120
Untreated bentonite	EE2	5.16	—	7	25	—	Chemisorption	LI and DM	PSO	132
Na-bentonite	EE2	5.21	—	7	25	—	Chemisorption	LI and DM	PSO	132
Trp-Na-bentonite	EE2	7.40	—	7	25	—	Chemisorption	LI and DM	PSO	132
Fe–Na- bentonite	EE2	6.28	—	7	25	—	Chemisorption	LI and DM	PSO	132
Fe-MIL-101-NH_2_	E2	202.02	—	—	37	—	H-bonding and π–π interaction	LI	PSO	133
	EE2	229.89	—	—	37	—	H-bonding and π–π interaction	LI	PSO	
	E3	205.85	—	—	37	—	H-Bonding and π–π interaction	LI	PSO	
Carbonized montmorillonite/carboxymethyl cellulose	E2	138.95	—	5	25	480	H-Bonding and π–π interaction	FI	Ritchie nth-order kinetic model	104
							Pore-filling			
							Hydrophobic partitioning			
							van der Waals interaction			
Lotus seedpod BC	E2	147.12	—	—	17	1200	Chemisorption	LI	PSO	90
							Electrostatic and π–π interaction			
K_2_FeO_4_ modified Lotus seedpod BC	E2	121.18	—	—	35	240	Micropore filling	LI	PSO	121
							Electrostatic and π–π interaction			
							Chemisorption			
Fe–Mn binary oxide/MWCNT	E2	47.25	86.16	7	25	360	H-Bonding and π–π interaction	LI	PSO	122

a Where AT = adsorption time, OT = optimum temperature, OP = optimum pH, HAE = highest adsorption efficiency, HAC = highest adsorption capacity, ASE = adsorbed steroidal estrogens, FI = Freundlich isotherm, LI = Langmuir isotherm, PFO = pseudo-first-order, PSO = pseudo-second-order, DM = dual mode AC = activated carbon, BC = biochar, HC = hydrochar, NP/NPs = nanoparticle(s), SWCNTs = single-walled carbon nanotubes, CNTs = carbon nanotubes, MWCNTs = multi-walled carbon nanotubes, rGO = reduced graphene oxide, GO = graphene oxide, HEMA = 2-hydroxyethyl methacrylate, MAPA = *N*-methacryloyl-l-phenylalanine, UF = ultra-filtration, and * = SE in multicomponent system. HAC was taken from LI.

**Fig. 8:**
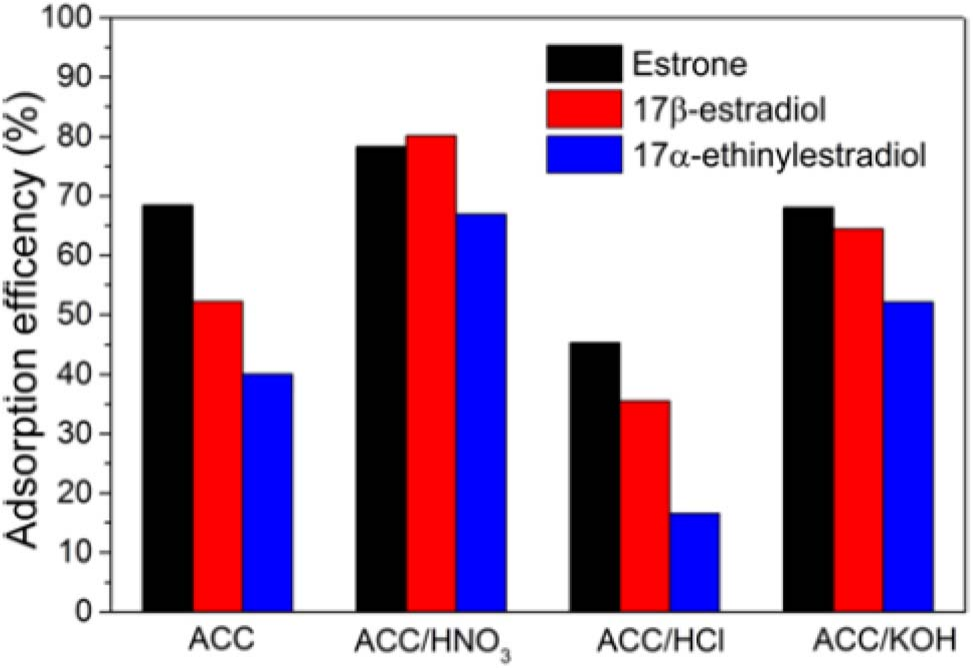
Adsorption efficiency of unmodified and chemically modified AC clothes.^73^ Reused with permission from Springer (order number: 501826394).

For instance, Prokić *et al.*^73^ comparatively examined the adsorption of three SEs from water using unmodified and chemically modified AC clothes (ACC). From the study, applied chemical (HNO_3_, HCl, and KOH) modification improved the specific surface area and the content of architectural oxygen functional moieties of the ACC, which elevate its adsorption capacity up to 30% with the one modified with HNO_3_, resulting in the highest adsorption efficiency for E1, E2, and EE2, as shown in Fig. 8. The adsorption followed PSO and fitted well with the Freundlich isotherm model, which models the physisorption mechanism.^73^ The remarkable adsorption efficiency (>80%) recorded for the ACC (HNO_3_) was consistent with 83.1% recorded for the removal of EE2 using hydrothermally carbonized and steam AC fabricated from palm kernel shells by.^68^

Although^68^ reported PSO kinetics in their research, the thermodynamic result demonstrates that the adsorption operation was predominantly chemisorption, which somewhat agrees with the report on the adsorption of E2 using magnetic molecularly imprinted polymers^74^ and 3-D covalently linked GO and rGO-methoxyl ether polyethylene glycol functionalized silica^75^ but differs from ^73^ that claimed physisorption. Furthermore, Akpotu *et al.*^75^ empirically pointed out that the −ve Δ*G*° obtained proved the spontaneity and thermodynamic propitiousness of the adsorption course, which improved with an uptick in SE temperature, while the +ve Δ*H*° and Δ*S*° figures obtained for the adsorbing material illustrate that the adsorption operation was endothermic and improved randomness/disorderliness between the adsorbent/adsorbate interfaces.

Additionally,^76^ reported the performance of single-walled carbon nanotubes (SWCNTs) for the adsorption of EE2 from seawater and brackish water. A 95% efficiency recorded was attributed to hydrophobic interactions and π–π electron (π–π e^−^).^76^ The adsorbent performance of SWCNTs is in a very close range with 93% reported for the adsorption of E2 using magnetic molecularly imprinted polymers fabricated on the surface of Fe_3_O_4_.^74^ This mechanism is consistent with those cited in several previous adsorption studies^77–81^ using CNTs, which considered a large specific surface area with evenly distributed hydrophobic sites for organic contaminant adsorption.

**Fig. 9:**
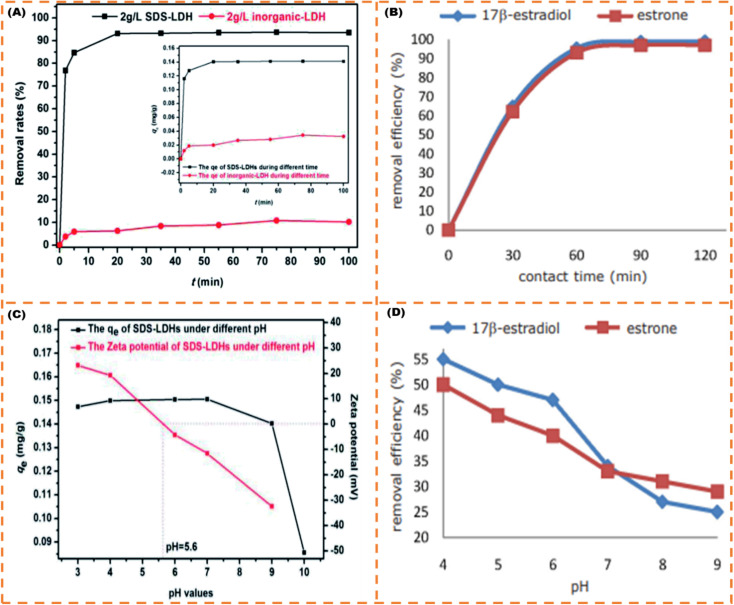
Contact time effect (A) and pH effect (C) on the removal efficiency of E2,^83^ contact time effect (B) and pH effect (D) on the removal efficiency of E1 and E2.^82^ (Open access).

Similar to the high adsorption efficiency reported in the foregoing paragraph,^82^ used rice husk silica also achieved excellent adsorption of 93.10% and 95.50% for E1 and E2 in 1 h at pH 4, respectively. It is noted that the adsorption efficacy of E1 and E2 reduced with an uptick in pH from 4 to 9 because with an upsurge in ambient pH, the composition of hydroxyl ions surges in the environment. Because the exterior surface of the adsorbing material has a +ve charge, the silica tends to absorb the copious volume of O_2_; thus, hydroxyl ions are adsorbed on the adsorbent at alkaline pH, which decreases the sorption capacity, *i.e.* the hydroxyl group present on the surface of the rice husk silica makes it −ve and creates a repulsive force between the adsorbing material and SE anionic molecules. As shown in Fig. 9, the dependency of adsorption efficiency on pH is parallel to that expounded by.^83,84^ Both teams^82,83^ also alluded to the fact that as the reaction time increases, the adsorption efficiency also increases and does not differ from what^85,86^ opined in their studies. Abdel-Gawad and Abdel-Aziz^86^ further investigated the effect of stirring on adsorption efficiency, and they observed that the percentage adsorption (84–85%) did not considerably change when the stirring tempo was increased from 200 to 500 rpm compared with 100 rpm (80%) even though the EE2 diffusion to the surface of the AC-alginate biopolymer was accelerated by the amplified stirring frequency. Notably, according to,^85^ the LI's *q*_max_ revealed that the E3 was the most temperature-sensitive adsorbate, with a reduction of 76.92% at 35 °C and 56.59% at 45 °C relative to the E3's *q*_max_ at 25 °C. For the E2, the *q*_max_ declined from 97.82% at 35 °C to 80.96% at 45 °C, but the E1 showed negligible sensitivity to the temperature upswing, exhibiting *q*_max_ of 98.85% at 35 °C and 96.60% at 45 °C. Additionally, the multi-component system (E1 + E2 + E3) exhibited similar traits displayed for the monocomponent system (Individual SE) as per the adsorption isotherm with a very close range of adsorption capacity (Table 1) under the same experimental conditions.^85^

To corroborate the unravelling of the mystery of the dependency of SE adsorptive operation on pH accentuated in the foregoing paragraph, Jiang *et al.*^87^ pointed out that there are three plausible explanations for how pH affects adsorption: (i) uptick in pH inhibits the formation of H-bond owing to the cleavage of functional groups; (ii) elevating pH might lead to an upsurge in the breakdown of the hydrophobic neutral SE into hydrophilic SE and then stimulate water cluster materialization around the polar spots of adsorbing material, leading to a reduction in hydrophobic interaction; and (iii) by increasing pH, various charged SEs would repel one another more electrostatically and limit/weaken the adhesion between them and the adsorbing material. In addition, most reports in Table 1 empirically confirm that the number of electrostatic charges, which the ES contributes to the adsorption process, is strongly governed by pH. This can be easily explained using pHzc (when the surface of the adsorbent material is uncharged). In other words, there is no net charge associated with the functional group charge.^88–91^ For example, as the pH of the solution increases, the surface potential becomes highly negative (when the pH is higher than pHzc), intensifying the electrostatic effect. When the pH falls below pHzc, the surface charge turns positive, making it challenging to adsorb positively charged SE molecules.^88,91,92^

**Fig. 10:**
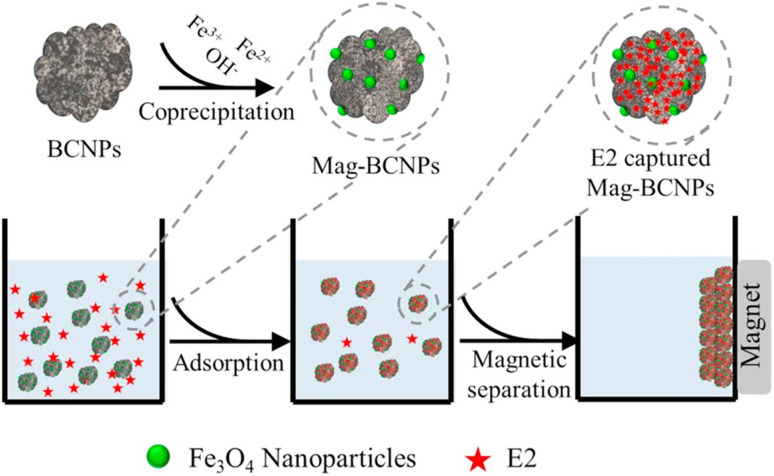
E2 adsorption process and magnetic separation.^93^ Reused with permission from Elsevier (order number: 557491148583).

**Fig. 11:**
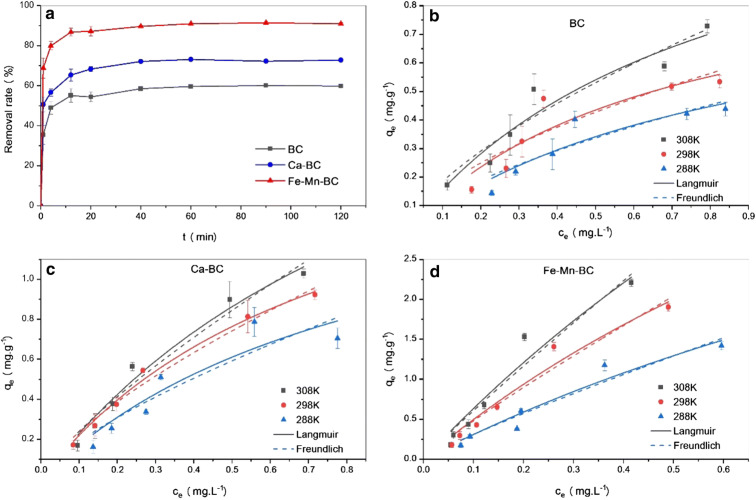
Adsorption performance and kinetic characteristics of modified and unmodified *Litchi chinensis* Sonn. BC for the aqueous removal of E1.^95^ Reused with permission from Springer (order number: 501826371).

**Fig. 12:**
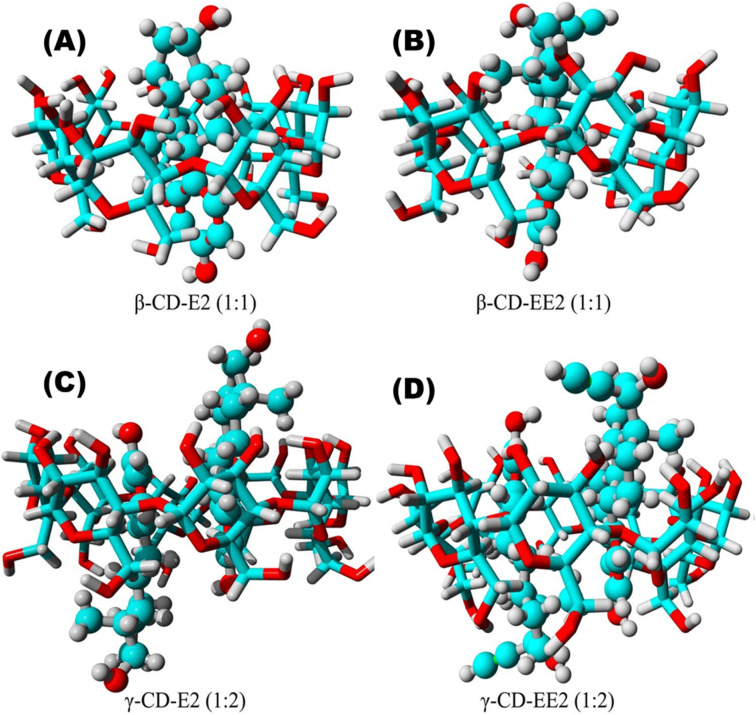
Models of the (A) β-cyclodextrin polymers-E2, (B) β-cyclodextrin polymers-EE2, (C) γ-cyclodextrin polymers-E2, and (D) γ-cyclodextrin polymers-EE2 complexes.^97^ Reused with permission from Elsevier (order number: 5577501114986).

Dong's team^93^ magnetically modified BC was employed for the removal of E2. The improved BC yielded excellent E2 adsorption with exceptional magnetic separation capability and powerful magnetic responsivity, as shown in Fig. 10. It was empirically established that the installation of Fe particles on the surface of BC provided supplementary binding spots, which in turn enabled efficient and quick access of E2 to the sorption spots and thus resulted in effective estrogen uptake in a shorter time compared to that reported by Liu *et al.*^94^ and Yin *et al.*^88^ using magnetically modified rice straw BC and its analogues supported by graphene oxide, resulting in effective estrogen uptake in 20 h and 5 h, respectively. However, they^88,94^ affirmed that the sorption efficiency of modified BC was much higher compared to that of unmodified BC. A similar scenario was observed for the removal of E1 using unmodified *Litchi chinensis* Sonn BC and its modified Ca and Fe–Mn-impregnated analogues.^95^ Notably, as shown in Fig. 11, both the modified and unmodified BC showed parallel adsorption kinetic profiles for the removal of E1, but the best adsorption efficiency of 91.50% was achieved by Fe–Mn BC in 2 hours, which was much better than the one achieved by BC (60.1%) and Ca-BC (73.2%). It was accentuated that Fe and Mn can oxidize the reducing entities on the BC surface, which may be the main reason for their high surface area and more adsorption sites, yielding outstanding performance^95,96^ in their SE adsorption experiment used *N*-propyl functionalized spherical mesoporous silica to accomplish improved and selective sorption of E1, E2, and EE2. In this study, apart from the fundamental function of hydrophobic interaction, the input of the carbonylic lone pair e^−^ on Carbon 17 of E1 was pragmatically confirmed to create stronger H-bonding with silicon OH and boosted the dipole–dipole interaction between E1 and the adsorbent than E2 and EE2. In addition, from another study, it was clear that E2 had a better adsorption capacity than EE2 using graphene nanosheets as the adsorbent, which might be strongly linked to E2's stronger hydrophobicity than EE2's.^87^ A somewhat synonymous greater adsorption efficiency of E2 than EE2 was observed when β-cyclodextrin polymers and γ-cyclodextrin polymers were used as adsorbents for rapid endothermic adsorptive operation.^97^ Compared to most adsorbents reported in Table S4,† both β-cyclodextrin and γ-cyclodextrin polymers came to the fore because of their high adsorption capacity and jaw-dropping removal efficiency. Such strong sorption capability could be attributed to the mesoporous configuration of the polymer and the exceptional packing ability of cyclodextrin, which can stimulate or help the SE to be implanted into the cyclodextrin hollow swiftly and form the host–guest inclusion complex, as shown in Fig. 12.^97^

**Fig. 13:**
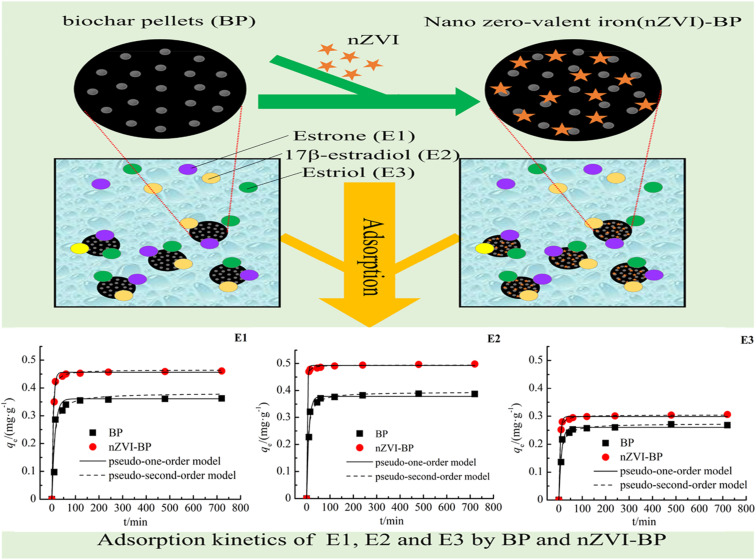
Adsorption kinetics of E1, E2, and E3 by BP and nZVI-BP.^99^ (Open access).

Kireç research group^98^ synthesized l-phenylalanine-containing poly(HEMA-MAPA) microparticles by microemulsion polymerization and utilized it as an adsorbent for the adsorption of E2 from aqueous solution. The microparticle adsorbent was reported to have a good adsorption capacity of 98.4 mg g^−1^ for E2 through hydrophobic interaction, while the adsorption operation fitted the Langmuir isotherm than Freundlich and Temkin. It was observed that the adsorption mechanism has a single layer and uniform surface that occurs spontaneously through physisorption, and the amount of adsorption increases as the temperature and ionic intensity increase.^99^ differs in their report as it was observed that the sorption mechanism of E2 and its collimates (E1 and E3) occur spontaneously through the chemisorption and fit well with Freundlich than Langmuir isotherm when nanoscale zero-valent iron-supported biochar pellets were used as the adsorbing material. More specifically, as shown in Table 1 and Fig. 13, the nZVI-BC pellet performed better than the BC pellet with E2 coming to the fore, followed by E1 and E3 based on the adsorption capability of the two adsorbing materials.^99^

**Fig. 14:**
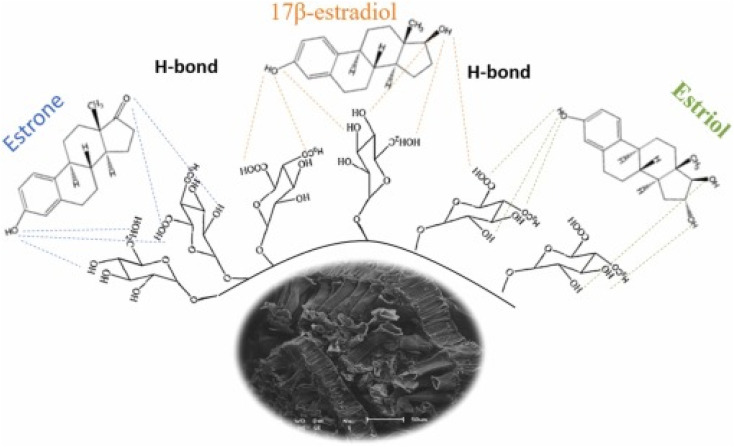
H-Bonding interaction of E1, E2, and E3 with the biosorbent (soybean hull).^100^ Reused with permission from Elsevier (order number: 5577511310171).

Honorio *et al.*^100^ explored the adsorption performance of soybean hull biosorbent for the adsorption of steroidal estrogen (E1, E2, and E3) in both single component (SC) and multiple components (MC (E1 + E2 + E3)) systems from swine manure (biofertilizer) effluent. From the adsorption evaluation, the best SE removal was performed at 25 °C and pH 7. For the SC and MC systems, equilibrium was attained in 60 minutes for E1 and 120 minutes for E2 and E3, and the PSO model did a good job of describing the kinetics. Models of MC equilibrium suggested that there was no contest between the steroidal estrogens. The optimum adsorption capabilities using LI for E1, E2, and E3 in the MC system were 2.560 mg g^−1^, 1.978 mg g^−1^, and 0.835 mg g^−1^, respectively. This is relatively in a very tight range with adsorption capacity recorded in SC, as shown in Table 1. Thermodynamically, the adsorption was physisorption in nature and favourably spontaneous through H-bonding, as depicted in Fig. 14. Honorio *et al.*^101^ achieved analogous performance in the E1, E2, and E3 adsorption using rice husk adsorbent and reported no contest between the SE in the SC and MC systems, within comparable equilibrium times (60 minutes for E1 and 120 minutes for E2 and E3), and this is closely consistent with the adsorptive removal of E1 and E2 in MC system investigated using Macadamia nutshell AC within an equilibrium time of 90 min and adsorption capacity of 22 mg g^−1^.^102^ Elias *et al.* and Honorio *et al.*^100,102^ empirically pointed out that E1 has a higher interaction capacity owing to the two e^−^ pairs available to generate H-bonding shown in Fig. 14 with the biomass OH functional groups, while E2 and E3 have a poorer interaction ability owing to the protonated active spot. Investigators^100,102^ also underscore that the E3 adjacent OH groups perhaps thwart the interaction with biomass compared to E1 and E2. However, Elias *et al.*^102^ differ by reporting that both LI and FI suit the experimental data of E1 and E2 adsorbed by nutshell AC, and this event was attributed to the complex mechanism. In addition, the E1 adsorption by few-layered boron nitride nanosheets^103^ and E1 and E3 adsorption onto a mesoporous molecularly imprinted polymer^91^ presented thermodynamic characteristics in parallel to that of.^100^ The authors^91,100,102,103^ reported that in physisorption, where the adsorption is caused because of the electrostatic interactions, the Δ*G*° is around −20 kJ mol^−1^, while in chemisorption, the Δ*G*° ranges from −84 to −40 kJ mol^−1^ and adsorption occurs because of e^−^ transfer, e^−^ exchange or e^−^ sharing.^100,102^ further accentuated that owing to the hydrophobic profile of SE, during the sorption operation, there is an imbalance between the surface of the adsorbent and its internal forces, fostering the operation to molecular adsorption by the van der Walls force among many other interactions reported in Table 1. Furthermore, according to Ahmed *et al.*,^92^ during the adsorption process, the SE phenolic moiety can create a resonating stabilized configuration by transferring e^−^ to the benzene ring. The improved electron density of the benzene ring enables it to function as a powerful e^−^ giver; at highly acidic pH (pH ≤ 2), the hydroxyl and ketonic functional groups are protonated, and the SE is in their cationic form.^92^ The findings above corroborate the findings of other researchers who have equally accentuated that interactions occur between the phenolic moiety of SE and the e^−^ acceptor groups tied to adsorbent,^95,104,105^ and this results in efficacious adsorption.

Analogue to the SE adsorptive studies juxtaposed in the foregoing paragraph, Debs *et al.*^106^ and Zhong *et al.*^107^ evaluated the adsorption of E1 and E2 using yeast biomass from an ethanol industry and HDTMA-modified zeolites, respectively. The PFO model described the adsorption studies,^106,107^ and Langmuir models best represented the equilibrium data, which indicates that the biosorbent is dynamically regular and that the SE-biosorbent interactions are uniformly distributed to the surface of the biosorbent.^100,106,107^ Furthermore, contrary to some authors,^85,100–102^ no-competition reports exist on the multi-component system (E1 + E2 + E3/EE/EE2) adsorption capacity. Debs *et al.* and Zhong *et al.*^106,107^ reported mixed associative/competitive sorption.

Sobhanardakani team^108^ performed an empirical adsorptive study using CeO_2_ decorated on CuFe_2_O_4_ nanofibers. A remarkable adsorption efficiency of 97.50% was achieved at 50 °C as against 70.50%, which was achieved at 20 °C. This trend of an increase in temperature leading to an increase in adsorption efficiency is contrary to that of,^100–102^ where the studies by the authors agreed that the adsorption operation lost efficiency with an increase in temperature. Sobhanardakani team^108^ established that the enhancement of EE2 adsorption with an increase in temperature is attributed to the improvement of the interaction frequency between EE2 moieties, and the adsorption spots with temperature uptick reveals that EE2 adsorption onto nanofibers adsorbent an endothermic operation in contrast to the exothermic process reported by.^100–102^ Conversely, the isotherm study revealed that the Elovich model could best describe the equilibrium data, and the findings also demonstrated that the adsorption operation followed the LI and FI models.^108^ The maximum adsorption efficiency (∼98%) observed by^108^ is comparable to the one (99%) reported for EE2 by^109^ using mesoporous carbons and another one (99%) reported by Tagliavini *et al.*^110^ for the adsorption of E2 in cellulose ultrafiltration membrane setup coupled with polymer-based spherical AC produced by carbonization and steam activation of cross-linked polystyrene. However, the efficiency is higher than the one (50%) reported by^111^ using walnut shell BC and the one (<95%) reported by^112,113^ using cellulose nanofiltration and ultrafiltration membranes setup coupled with polymer-based spherical AC.

Martins *et al.*^114^ studied the use of physically modified and chemically activated sludge from a water treatment plant as an adsorbent for the removal of E2 and EE2. The effect of the two activating agents (KOH and H_2_SO_4_) used on the adsorptive capacity of the sludge adsorbent was comparatively explored with that of the physically modified (through heat treatment) one. E2 and EE2 gave better adsorption capacities at the lowest adsorbent dosage with a reduction in adsorption capacities as the dosage increased. It can, however, be noted that the chemically activated sludge stood out in the adsorption of both SEs when physically modified compared to the one activated with KOH coming to the fore in all, except for the 0.5 g dosage where H_2_SO_4_ managed to come to the fore for EE2 adsorption with varied equilibrium times between 180 and 420 min. It was holistically established that as the adsorbent dose increases, the availability of higher energy spots diminishes, with a greater percentage of low energy sites occupied, leading to a reduced *q*_e_ value. The smaller mass allows all active spots to be accessible and the adsorption on the surface to be promptly saturated. The increase in the diffusion route length caused by the overlapping of adsorption spots as a function of the increase in the adsorbent dose and the active spots that remained unsaturated following adsorption are additional factors in the decrease in *q*_e_.^114^ The adsorption equilibrium time obtained by ref. 114 is within the commonly reported range,^115–118^ while the isotherm model is similar to that of ref. 115 and 116. In addition, Yin research group^116^ observed a reduction in adsorption capacity with an increase in pH and examined the influence of various background electrolytes on the adsorption operation of E2 by adding different anions and cations under different concentrations into the reaction system. Attapulgite/BC was shown to have better adsorption efficacy when monovalent cations (Na^+^ and K^+^) were present, and its adsorption capacity only marginally diminished as the concentration increased from 0.01 to 0.1 g L^−1^. However, the ability of E2 to adsorb was hampered by the presence of divalent cations (Ca^2+^ and Mg^2+^). Owing to the strong polarization strength of the divalent cations, which was larger when they were present, the squeezing-out effect may be used to elucidate these phenomena. Moreover, owing to the effects of background electrolyte anions, such as Cl, NO_3_, SO_4_^2−^, and PO_4_^3−^, on E2 adsorption onto attapulgite/BC, anions were found to have no noticeable effects on the adsorption of E2 from an aqueous medium. The adsorption capacity of attapulgite/BC diminished marginally in the presence of anions. This behavior might be explained by the electrostatic attraction of anions to the −vely charged attapulgite/BC surface, which diminished the material's ability to adsorb E2.^116^ Other research groups^119–122^ also obtained in another study that the presence of the two cations is common in effluents (Ca^2+^ and Na^+^); effluents did not significantly influence SE removal efficiency when Fe_3_^+^-saturated montmorillonite, binary oxide of Fe–Mn/MWCNT, K_2_FeO_4_ modified Lotus seedpod BC and green tea bio-fabricated rGO@Fe NPs were employed. A few findings^18,90^ have also alluded to the fact that natural organic matter carries an overall −ve charge owing to a lot of carboxyl and phenolic moieties in its structure, which makes it exhibit a negligible competitive effect with infinitesimal variation in adsorption capacity^90,93,121,123^ or form interactions (NOM-SE and NOM-adsorbent) that considerably diminish the adsorption of SE; this is because of the polar functionalities that are generated on the surface of the BC following bonding with NOM, which stimulates the materialization of water clusters around the adsorbent surface through a far-reaching H-bonding network and leads to lose of adsorbent hydrophobicity and SE-adsorbent hydrophobic interaction.^94,95,123^

It is imperative to say that in our view, different adsorbing materials respond differently to different activating agents, and this accounts for variation in the adsorbing capacity/efficiency of different chemically activated adsorbents reported.

Going forward, the advent of the fabrication of biogenic NPs using biological entities (plant and microorganisms)^124–126^ has also resulted in jaw-dropping adsorption records of many organic pollutants, including steroid estrogens.

**Fig. 15:**
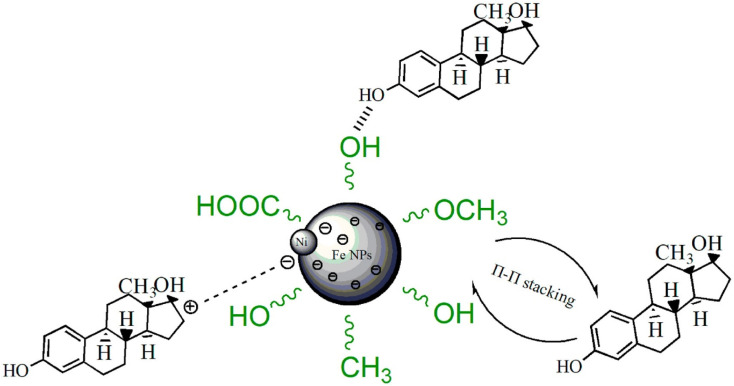
Possible mechanism of carbonized biogenic Fe/Ni NP combinatorial adsorption of E2.^127^ Reused with permission from Elsevier (order number: 5577531212233).

For instance, Gong *et al.*^127^ studied the use of carbonized green bimetallic Fe/Ni NPs biosynthesized using *eucalyptus* leaf extract to adsorb E2 from an aqueous solution. The carbonized biogenic Fe/Ni NPs efficiently adsorbed E2 in less than half an hour according to the batch adsorbent studies, with optimum adsorption performance of 98.3%. Compared to other adsorption efficiencies of other adsorbents reported in Table 1, this outstanding efficiency by the biosynthesized NPs was partly attributed to their improved specific surface area and better porous architecture following carbonization; the BET study revealed that the surface area of green Fe/Ni NPs improved from 31.99 m^2^ g^−1^ before carbonization to 57.57 m^2^ g^−1^ upon carbonization. In addition, as shown in Fig. 15, the surface of carbonized-Fe/Ni NPs still has many oxygen-containing functional groups derived from the bioactive compounds present in the *eucalyptus* leaf extract, which may have a combinatory effect to expedite better E2 adsorptive removal. Unfortunately, only 48.7% and 51.8% of E2 were removed from pig runoffs and domestic sewage, respectively, when the same novel biogenic NPs were applied in a real-life scenario. However, the authors^127^ justified this low removal percentage by considering the massive volumes of dissolved organic materials that are often present in raw effluents, which hypothetically influence the adsorptive removal of E2 in a negative way. This is due to the presence of highly electron-loaded functional groups, such as phenolic and aniline, in dissolved organic materials, resulting in a high propensity to participate in various environmental reactions and thus effortlessly react with the Fe^3+^ surface. Thus, one of the causes of the decrease in E2 adsorption effectiveness in both pig runoffs and domestic sewage compared to that reported for ultrapure water is the presence of dissolved organic matter in raw effluents.^127^ This justification is consistent with what was earlier established by Qin's group^119^ in their studies using Fe_3_^+^-saturated montmorillonite, even though the efficiency (93%) reported is less than the 98.30% and 99% reported for carbonized biogenic Fe/Ni NPs^127^ and green tea bio-fabricated rGO@Fe NPs, respectively.^120^

**Fig. 16:**
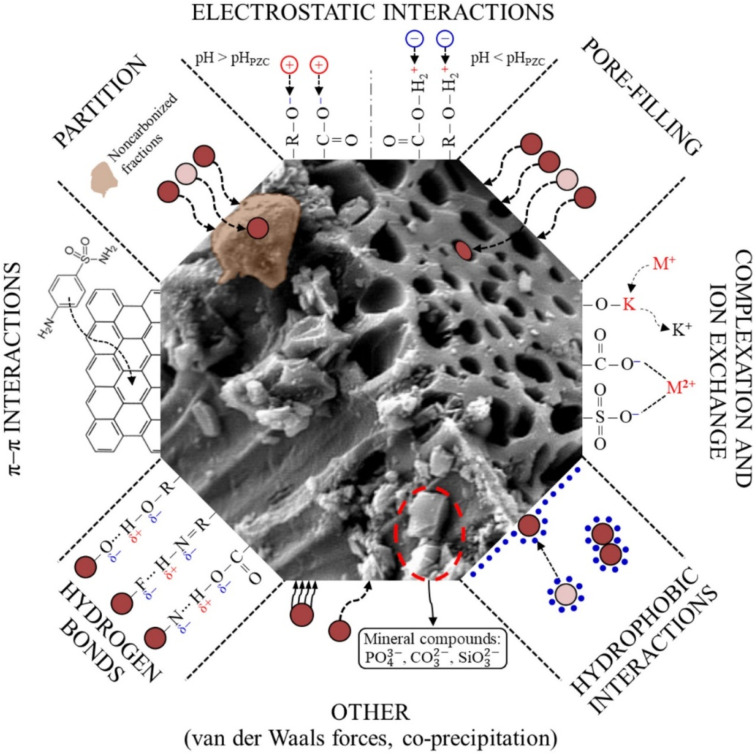
Plausible mechanistic adsorbent-SE interactions.^129^ Reused with permission from Elsevier (order number: 557740119124).

To summarise, as seen in the adsorption efficiency discussion in this concluded section, it is obvious that in the chemistry of SE adsorption, the major challenge is how to select the most promising types of adsorbents, as per low cost, benignness, high adsorption capacity, high adsorption rate, and rapid kinetics; to overcome these challenges, a grounded understanding of the SE adsorption mechanism is required. Moreover, understanding the SE adsorption mechanism offers a win–win insight into designing an outstanding desorption strategy for the recovery of adsorbed SE and the recyclability of spent adsorbents.^128^ However, in a bid to identify the SE adsorption mechanism(s) (particularly the interactions occurring at the adsorbent/SE interface) to easily overcome those aforementioned challenges and take the SE adsorption process to the next level, this also becomes another real challenge.^128^ In general, amid all the holistic and pragmatic explanations of SE adsorption presented in the foregoing paragraphs, its mechanisms are not fully understood because many interactions are possible, as shown in Fig. 16 (ref. 18, 88, 92, 93, 104, 105, 123) and Table 1. These interactions are largely governed by the experimental settings (temperature, kinetics, adsorbent dose, pH, and background electrolytes), the SE profile (mixability/dissolution rate, molecular weight, and p*K*_a_), and the adsorbent profile (surface architectural functional moieties and surface area). Thus, this conclusively left us with an intriguing query that overlaps with that of Crini *et al.*^128^ about whether all the aforementioned interactions have to be considered to fully comprehend the SE adsorption mechanism. In our view, this question's response is somewhat complicated. Depending on the adsorbent architectural makeup, the SE makeup and its characteristics, the solution pH, temperature, and ionic strength, it is feasible that more than one of these interactions can occur concurrently in the adsorption process of SE, as shown in the report of various literature presented in Table 1.

## 5. Desorption and reusability

Adsorbents after adsorbing contaminants may still be potentially harmful to the ecological system because adsorption is merely a physicochemical operation that encompasses the mass transfer of a solute from the liquid phase to the surface of the adsorbing material; thus, the regeneration and recyclability of spent adsorbent is a proficient mechanism towards achieving a practicable, reliable, effective, industrial and eco-economical SE adsorptive remediation technique.^75,93,108,120,134^ The regeneration entails removing spent adsorbent from the reaction mixture and then removing loaded SE from the surface using an eluting reagent or instrument, such as a centrifuge.^75,134^ An ethanol solution was employed as an eluting solvent by Beri *et al.*^68^ to remove EE2 from palm kernel shell AC. The palm kernel shell AC was recovered from the regeneration solution by filtration, washed with water, and dried at 80 °C for 720 minutes. The number of adsorption–regeneration cycles was used to test reusability. The result revealed that the regeneration process was not effective enough, and the desorption of EE2 reduced to 32% after four cycles.^68^ This significant reduction in adsorption capacity is in parallel with what was reported when distilled water and acetonitrile were used in four cycles of adsorption–desorption of rice husk silica and E1 and E2.^82^ The high activation energy (60.4 kJ mol^−1^) of EE2 sorption, which suggests that chemisorption is the primary process occurring on the palm kernel shell AC adsorbent, may be used to justify the dramatic decrease in the EE2 sorption process after the fourth cycle. Notably, according to the authors,^68^ chemisorbed SE is considerably tougher to desorb from the absorbing material than a physisorbed one. However, it was opined that increasing the temperature of the regeneration process should enhance the removal of EE2.^68,74^ explored a regeneration study on the E2 loaded on Fe_3_O_4_@SiO_2_@MPS by applying different eluting solvents, such as methanol, ethanol, acetone, and acetonitrile. It was observed that the optimal removal of E2 was at best for methanol, followed by ethanol, then acetonitrile and acetone. It was then observed for the methanol desorption experiment that the binding ability of Fe_3_O_4_@SiO_2_@MPS for E2 hardly decreased after about five times of reuse.

In another study by Tang *et al.*,^97^ after six cycles of using the β-cyclodextrin polymer and γ-cyclodextrin polymer adsorbents, the desorption proficiency of the regenerated β-cyclodextrin polymer and γ-cyclodextrin polymer was sustained above 99%, and almost no reduction was witnessed after five adsorption-regeneration successions, thereby making β- and γ-cyclodextrin polymers appropriate and efficacious polymeric adsorbing materials for E2 and EE2 removal from aqueous solutions.

Furthermore, magnetization is a value-added strategy that is included for the simple reclamation of the used adsorbing material. In various studies, magnetically engineered adsorbents have been employed by researchers for the sorption of SE and have shown remarkable reusability.^88,93,94^ For example, in a study conducted by Dong's team,^93^ ozone treatment, which broke down the adsorbed E2 on the surface of adsorbents, was used to regenerate BC/Fe_2_O_3_NPs. Each cycle involved the reaction of 1 mg L^−1^ of E2 and 0.1 mg mL^−1^ of BC/Fe_2_O_3_NPs for 25 min at 25 °C. BC/Fe_2_O_3_NPs were retrieved by magnet separation after reaching adsorption equilibrium. This was done after purging with an ozone gas (10 g h^−1^) for 10 minutes and washing with deionized water three times. The subsequent E2 adsorption process by the renewed BC/Fe_2_O_3_NPs required the addition of a new E2 solution. As shown in Table 2, there was no considerable reduction in the sorption efficiency of the BC/Fe_2_O_3_NP adsorbent after five sorption-regeneration series. A similar insignificant diminution in the sorption efficiency of adsorbents after five adsorption-regeneration cycles was observed by other researchers.^88,108,109,116^Table 2 provides an overview of desorption and recycling in the available studies. Various eluting reagents, such as NaOH,^18^ HCl,^108^ HNO_3_,^116^ ethanol,^74,94,109,116^ methanol,^74,91^ acetic acid,^91^ acetonitrile,^74,82^ and acetone^74^ have been used to desorb different SEs from spent adsorbing materials.

**Table 2:** Summary of steroidal estrogen (SE) desorption and adsorbent reuse

Adsorbent	SE	Eluent	% Adsorbed (at *n* = 1)	No. of cycles	% Adsorbed after *n* cycles	References
BC/Fe_2_O_3_NPs	E2	Magnet + ozone gas	>90	5	89.26	93
SBA-rGO-mPEG	E2	acidic ethanol (adjusted with 0.1 M HCl)	90	4	86	75
Palm kernel shells AC	EE2	20 mL of 4 mg^−1^ of ethanol	54.60	5	32.30	68
Fe_3_O_4_@SiO_2_@MPS	E2	10 mL methanol	98	5	>85	74
Rice husk silica	E1	Double-distilled water and acetonitrile	93.10	4	5	82
	E2	Double-distilled water and acetonitrile	95.50	4	3.5	
GO-magnetic rice straw BC	E2	100 mL ethanol	100	5	87	94
Activated magnetic rice straw BC	E2	100 mL ethanol deionized water	100	5	>80	88
β-Cyclodextrin polymers	E2	10 mL ethanol	>99	5	>99	97
	EE2	10 mL ethanol	>99	5	>99	
γ-Cyclodextrin polymers	E2	10 mL ethanol	>99	5	>99	97
	EE2	10 mL ethanol	>99	5	>99	
Poly(HEMA-MAPA) microparticles	E2	Acetonitrile : methanol (70 : 30; v/v)	—	10	92.70	98
Mesoporous imprinted polymer	E1	1.25 mL of MeOH : acetic acid (9 : 1, v/v)	100	5	84.60	91
	E3	1.25 mL of MeOH : acetic acid (9 : 1, v/v)	100	5	84.6	
CeO_2_/CuFe_2_O_4_ nanofibers	EE2	0.1 mol L^−1^ HNO_3_	97.50	6	>92	108
Mesoporous carbons	EE2	10 mL ethanol	100	10	>85	109
Attapulgite-rice straw BC	E2	100 mL ethanol	99	5	>89	116
		Milli-Q water				
rGO@Fe NPs (using green tea extract)	EE2	Centrifugation	99.40	5	64.40	120

## 6. Outlook

The adsorption technique for the removal of various contaminants, including steroid estrogens, in water is a very promising area that has been explored by several researchers. Research in this area could focus on developing sustainable adsorbents with enhanced selectivity, specificity and adsorption capacity for SE removal either by modifying the existing adsorbents to improve their properties or by synthesizing materials tailored towards SEs removal.

For future studies, an in-depth study is required to understand the mechanism involved in SE adsorption and to explore the potential of combining it with other water treatment techniques. Furthermore, there is a need to evaluate the practical feasibility and scalability of adsorption techniques for large-scale applications, cost-effectiveness, regeneration of spent adsorbents and the development of efficient adsorption systems for real-life water treatment applications.

Because the *in situ* implementation of the commonly used chemical regeneration method is not yet feasible, the management of the eluted product during the chemical regeneration of the spent adsorbents should be considered for total environmental security.

## 7. Conclusion

This systemic scientometric review provided a comprehensive analysis of the sequestration of SEs in aqueous samples using the adsorption mechanism by examining and analyzing a wide range of scientific publications (articles, review articles, and book chapters) on SE removal from the Web of Science (WoS) database from January 1, 1990, to November 5, 2022, and the Scopus database from January 1, 1949, to November 5, 2022, which are leading citation databases. A total of 137 documents was used to methodically map bibliometric indicators, such as the number of articles, most prolific countries, most productive scholars, and most cited articles. It emphasizes the diverse range of adsorbents utilized, including activated carbon, zeolites, biochars, and MOFs, revealing the potential of these adsorbents in efficiently removing SEs and mitigating their adverse effects on human health and aquatic ecosystems. Various factors influencing the adsorption process, such as the initial concentration, pH, temperature, and physicochemical properties of the adsorbent, were studied to decipher the adsorption mechanism. The mechanisms are mostly observed to be physisorption, hydrophobic interaction, π–π interaction, H-bonding, chemisorption, or pore filling. One significant relevance accrues to the use of adsorbents is the desorption and reusability concept, which involves the regeneration of the spent absorbent by removing the loaded SEs on the surface using an eluting agent or centrifuge. Overall, the sequestration of SEs through adsorption is a promising approach with significant potential for environmental remediation and also helps researchers with an advanced understanding of refined adsorption techniques that will contribute to the protection of water resources and the wellbeing of the human population and ecosystem.

## Abbreviations

SEs: Steroid estrogens
EDC: Endocrine-disrupting chemicals
WHO: World Health Organization
E1: Estrone
E2: 17-β-Estradiol
E3: Estriol
EE2: 17-α ethinylestradiol
(*K*_ow_): Partition coefficient
NOEL: No-observed-adverse-effect
SDG: Sustainable development goals
OPEC: Organisation of petroleum exporting countries
WoS: Web of science
GO: Graphene oxide
CNT: Carbon nanotube
rGO: Reduced graphene oxide

## Author contributions

Ajibola. A. Bayode: conceptualization, supervision, software, writing – original draft, review, and editing. Chijioke Olisah: writing – original draft, software, review, and editing. Stephen Sunday Emmanuel: writing – original draft, review, and editing. Morenike Oluwabunmi Adesina: writing – original draft. Koko Daniel Terlanga: writing – original draft.

## Conflicts of interest

There are no conflict to declare.

## Supplementary Material

RA-013-D3RA02296J-s001
