# Supplementary material for: Sequestration of steroidal estrogen in aqueous samples using an adsorption mechanism: a systemic scientometric review
Source: RSC Adv. 2023 Jul 26;13(33):22675–97. doi: 10.1039/d3ra02296j (PMC10369132; doi:10.1039/d3ra02296j)
Supplement: RA-013-D3RA02296J-s001 [file RA-013-D3RA02296J-s001.pdf]

Electronic Supplementary Material (ESI) for RSC Advances.
This journal is © The Royal Society of Chemistry 2023

The Sequestration of Steroidal Estrogen in Aqueous Samples by Adsorption Mechanism: A Systemic Scientometric Review

‡Ajibola A. Bayode[1], Chijioke Olisah[2], Stephen Sunday Emmanuel[3], Morenike Oluwabunmi Adesina[4], Koko Daniel Terlanga[1]

[1]Department of Chemical Sciences, Faculty of Natural Sciences, Redeemer's University, P.M.B. 230, 232101, Ede, Nigeria

[2] Institute for Coastal and Marine Research, Nelson Mandela University, P.O Box 77000, Gqeberha 6031, South Africa

[3]Department of Industrial Chemistry, Faculty of Physical Sciences, University of Ilorin, P.M.B. 1515, Ilorin, Nigeria

[4]Department of Chemistry, Faculty of Natural and Applied Sciences, Lead City University, Nigeria

SUPPLEMENTARY INFORMATION

Table S1: Summarized bibliographic data.

**General bibliometric information**	
Timespan	1973: 2022
Sources (Journals, Books,	75
Documents	137
Average years from publication	5.51
Average citations per document	28.72
Average citations per year per doc	3.691
References	5695
**Document Types**	
Article	131
Book chapters	2
Review	4
**Document Contents**	
Keywords plus (ID)	1127
Author's keywords (DE)	417
**Authors**	
Authors	476
Author appearances	672
Authors of single-authored documents	0
Authors of multi-authored documents	476
**Authors Collaboration**	
Single-authored publication	0
Publication per Author	0.288
Authors per publication	3.47
Co-Authors per publication	4.91
Collaboration index	3.47

Table S2: Most productive countries on studies involving the removal of SEs from a contaminated environment.

Most productive countries								Total number of citations per country			
Rank	Countries	Articles	% Of 137	Freq	SCA	MCA	MCA/A Ratio	Rank	Country	Article citations	Citation average
1	China	47	34.31	0.35	44	3	0.06	1	China	1172	24.94
2	Brazil	17	12.41	0.13	15	2	0.12	2	USA	442	73.67
3	Iran	11	8.03	0.08	11	0	0.00	3	Spain	357	357.00
4	India	8	5.84	0.06	7	1	0.13	4	India	342	42.75
5	USA	6	4.38	0.04	4	2	0.33	5	United Kingdom	325	162.50

6	Czech Republic	5	3.65	0.04	4	1	0.20	6	Brazil	204	12.00
7	Malaysia	4	2.92	0.03	1	3	0.75	7	Japan	177	177.00
8	Austria	3	2.19	0.02	2	1	0.33	8	France	128	42.67
9	France	3	2.19	0.02	1	2	0.67	9	Singapore	103	51.50
10	Italy	3	2.19	0.02	3	0	0.00	10	New Zealand	100	50.00
11	Serbia	3	2.19	0.02	3	0	0.00	11	Iran	87	7.91
12	South Africa	3	2.19	0.02	2	1	0.33	12	Malaysia	70	17.50
13	Turkey	3	2.19	0.02	3	0	0.00	13	Austria	63	21.00
14	New Zealand	2	1.46	0.01	1	1	0.50	14	Italy	44	14.67
15	Singapore	2	1.46	0.01	1	1	0.50	15	Sweden	42	21.00
16	Sweden	2	1.46	0.01	2	0	0.00	16	Korea	24	24.00
17	United Kingdom	2	1.46	0.01	2	0	0.00	17	Czech Republic	22	4.40
18	Egypt	1	0.73	0.01	1	0	0.00	18	South Africa	21	7.00
19	Ethiopia	1	0.73	0.01	0	1	1.00	19	Greece	20	20.00
20	Finland	1	0.73	0.01	1	0	0.00	20	Serbia	15	5.00

SCA – Single country article; MCP – multiple country article

Table S3. The ten most cited articles on SEs removal research were retrieved from the Scopus and WoS databases.

Rank	First Author Names, Initials and Publication Year	Article Title	Journal	TC	TC/Y
1	Pan B, 2008	“Adsorption and hysteresis of bisphenol A and 17α-ethinyl estradiol on carbon nanomaterials”	Environ Sci Technol	366	24.4
2	Zhang Y, 2005	“Removal of estrone and 17β-estradiol from water by adsorption”	Water Res	172	9.56
3	Chen CY, 2007	“Determining estrogenic steroids in Taipei waters and removal in drinking water treatment using high-flow solid-phase extraction and liquid chromatography/tandem mass spectrometry”	Sci Total Environ	151	9.44
4	Ali I, 2017	“Supra molecular mechanism of the removal of 17-β-estradiol endocrine	J Mol Liq	137	22.83

		disturbing pollutant from water on functionalized iron nano particles"			
5	Jiang L, 2017	"Adsorption of estrogen contaminants by graphene nanomaterials under natural organic matter preloading: comparison to the carbon nanotube, biochar, and activated carbon"	Environ Sci Technol	119	19.83
6	Rovani S, 2014	"Development of a new adsorbent from agro-industrial waste and its potential use in endocrine disruptor compound removal"	J Hazard Mater	83	9.22
7	Zhongbo Z, 2008	"Selective removal of estrogenic compounds by molecularly imprinted polymer (MIP)"	Water Res	72	4.8
8	Han J, 2013	"Adsorption of ethinylestradiol (EE2) on polyamide 612: Molecular modelling and effects of water chemistry"	Water Res	65	6.5
9	Tang P, 2019	"A simple and green method to construct cyclodextrin polymer for the effective and simultaneous estrogen pollutant and metal removal"	Chem Eng J	57	14.25
10	Tang P, 2018	"Rapid and efficient removal of estrogenic pollutants from water by using beta-and gamma-cyclodextrin polymers"	Chem Eng J	49	9.8
